# A fast topological analysis algorithm for large-scale similarity evaluations of ligands and binding pockets

**DOI:** 10.1186/s13321-015-0091-5

**Published:** 2015-08-20

**Authors:** Mohammad ElGamacy, Luc Van Meervelt

**Affiliations:** Department of Protein Evolution, Max Planck Institute for Developmental Biology, 72076 Tübingen, Germany; Biomolecular Architecture, Chemistry Department, KU Leuven, Celestijnenlaan 200F, box 2404, 3001 Leuven (Heverlee), Belgium

**Keywords:** Topology comparisons, Ligand-based design, Binding pocket mapping, Virtual high-throughput screening

## Abstract

**Motivation:**

With the rapid increase of the structural data of biomolecular complexes, novel structural analysis methods have to be devised with high-throughput capacity to handle immense data input and to construct massive networks at the minimal computational cost. Moreover, novel methods should be capable of handling a broad range of molecular structural sizes and chemical natures, cognisant of the conformational and electrostatic bases of molecular recognition, and sufficiently accurate to enable contextually relevant biological inferences.

**Results:**

A novel molecular topology comparison method was developed and tested. The method was tested for both ligand and binding pocket similarity analyses and a PDB-wide ligand topological similarity map was computed.

**Conclusion:**

The unprecedentedly wide scope of ligand definition and large-scale topological similarity mapping can provide very robust tools, of performance unmatched by the present alignment-based methods. The method remarkably shows potential for application for scaffold hopping purposes. It also opens new frontiers in the areas of ligand-mediated protein connectivity, ligand-based molecular phylogeny, target fishing, and off-target predictions.

**Electronic supplementary material:**

The online version of this article (doi:10.1186/s13321-015-0091-5) contains supplementary material, which is available to authorized users.

**Graphical abstract:**
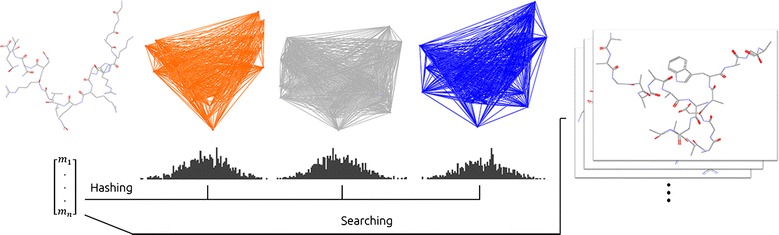
A novel molecular topology comparison method based on a combined shape distribution and charge binning scheme is presented.

## Background

Large scale analysis of biomolecular networks has proven to be of great utility in offering genuine insights into molecular interactions and construction of systematic inferences. For instance, this has been demonstrated in protein–protein interactions [1], disease underlying protein networks [2], protein binding pocket analysis [3], shared side-effects networks for drug target identification [4], protein–ligand phylogenetic analysis [5], cross-binding ligand networks [6], and even in network pharmacology [7]. Such analyses are normally based on a wealth of different types of data (e.g. sequence, structural, biochemical assays, chemical, etc.) or combinations thereof, and can provide highly refined, knowledge-based descriptions and models.

The rate of deposition of biomacromolecular structures in the Protein Data Bank (PDB) has been proven to exhibit an exponential growth [8, 9]. This was accompanied by a trending increase in average resolution and macromolecular complexes sizes. Consequently, there is a need for the design of computational tools and analysis methods capable of efficiently processing such volumes of structural data. This could ultimately enable the construction of large scale networks based on the whole repertoire of tertiary and quaternary structures.

The aim of this study was to introduce a new method capable of conducting large scale topology analyses of molecular structures that is specially suited for handling large and complicated topologies while being insensitive to minor conformational changes. As the molecular topology is defined here as the 3D structure and its associated electrostatic landscape, a method capable of effectively including the topology information should have a wide variety of applications. Herein, we investigate two particular applications to demonstrate such diverse applicability: ligand-based and binding pocket-based topology analyses. The aim was the development of a highly efficient fingerprinting method given the scale of the problem per se (given the expanse of data at the set definitions) and the structurally diverse nature of the envisaged purposes. Thus, a novel means of high-throughput topological analysis was devised and the algorithmic details of which are outlined below.

Molecular recognition events are at the core of almost every biological process, and are meant to remain an active area of research in both basic and applied realms. Therefore, the ability of reading through and topologically analysing the huge arrays of structural data available comes of a great significance for mapping ligands or their binding cavities into large networks. These include, for example, the elucidation of protein–protein relations in terms of ligand or cavity topological similarity via quantitative measures and the prediction of unreported potential ligand-target interactions by detecting potentially cross-binding ligands. Also, extension of the latter for predicting off-target (polypharmacology and toxicity) interactions of a query ligand, or extension towards ligand-based drug design by inspecting topology similarity between a native ligand as query against a library are possible applications, or even ligand-based target fishing, using instead a single query ligand against the array of native ligands.

Conceptually, this can be done directly by comparing the ligand–ligand structural relations, or alternatively, through comparing the receptor–receptor structural relations in light of the information encoded in the corresponding binding pockets topologies.

Although the applications are not limited to the above mentioned, in this report, the aim was to emphasise the strength of such large scale analysis method and to demonstrate the capacity to provide a PDB-wide map of topological similarity at very low computational expenses as well as the robustness of the exact implementation in binding pocket classification.

## Results

### PDB data preparation

As the PDB coordinates files were split into their constituting molecules using OpenBabel, applying the criteria described in the “Methods” section, the number of structures obtained was 164,939 without removing any redundancy (as redundancy could be optionally removed downstream the computations upon results parsing), where self-matches indicate similarity between repeated PDB molecules with slightly different conformations. These molecules were the ones subject to the topological analysis.

### Case studies

Multiple cases were investigated for a critical assessment of the algorithm’s strengths and liabilities. This was done by running searches (against all of the ligands retrieved from the database) and analysing the top results for a number of diverse target-bound structures of different chemical natures, where the query structure and search space topologies were only obtained from the target-bound conformations in the PDB structure.

**Fig. 1:**
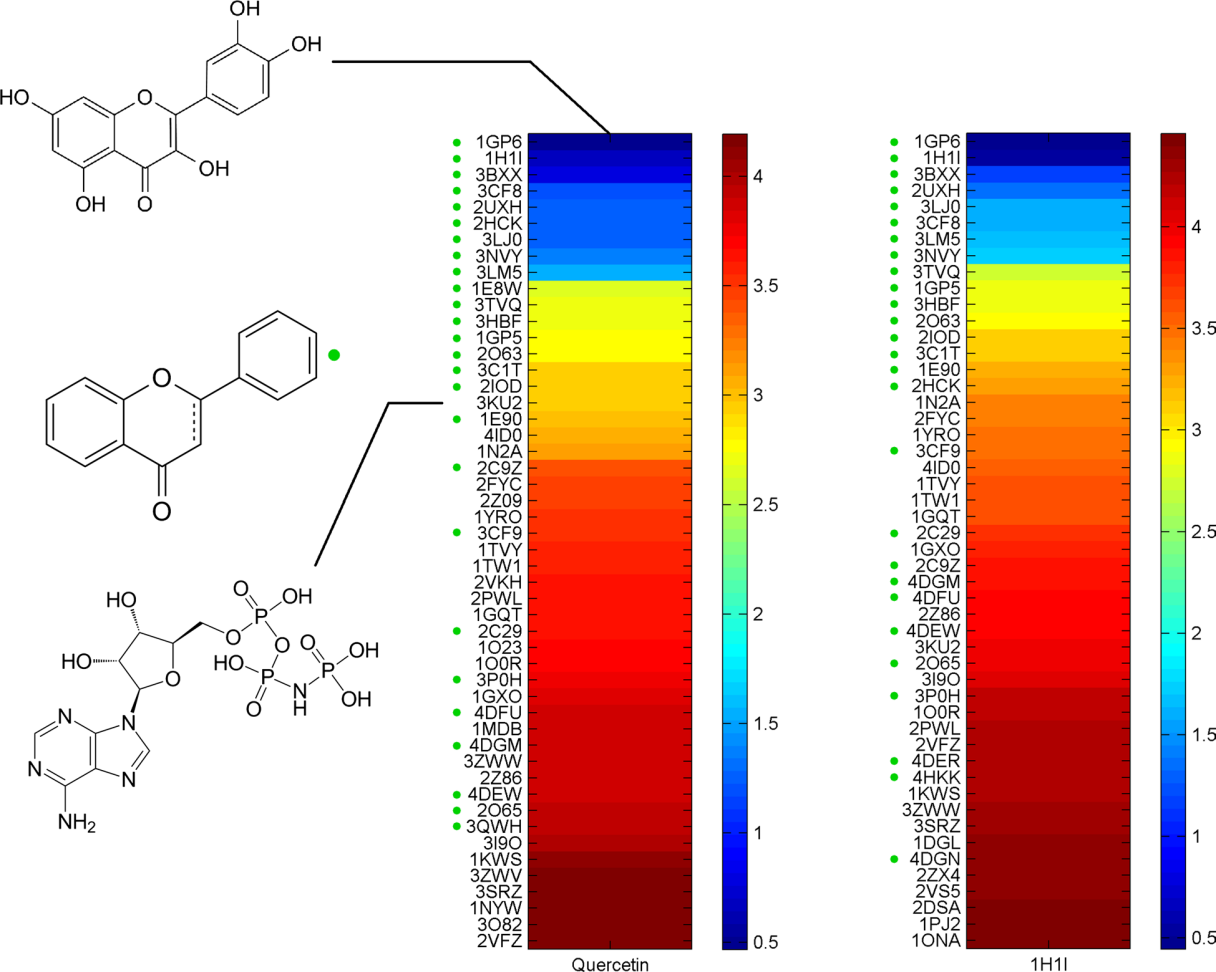
Two heat map strips of dissimilarity scores using the original conformation of quercetin from 1H1I (*right*), and six generated conformations (*left*) against the PDB ligands. The strips enlists the top 50 similar ligands (corresponding *colour legends* shown), *upper left* shows the chemical structure of quercetin, the *green dot* marks hits with flavonoid scaffold. The *colour legend* represents the dissimilarity score (*d*) scale (see “Methods” section).

First, two searches were conducted using as query the quercetin structure as obtained from the crystal structure of quercetin complexed with quercetin-2,3-dioxygenase (PDB: 1H1I) and six conformers sampled from the latter. Results were sorted according to the dissimilarity score in ascending order (Fig. 1). This was a control case with one rotatable bond to demonstrate the effect of using multiple conformers.

**Fig. 2:**
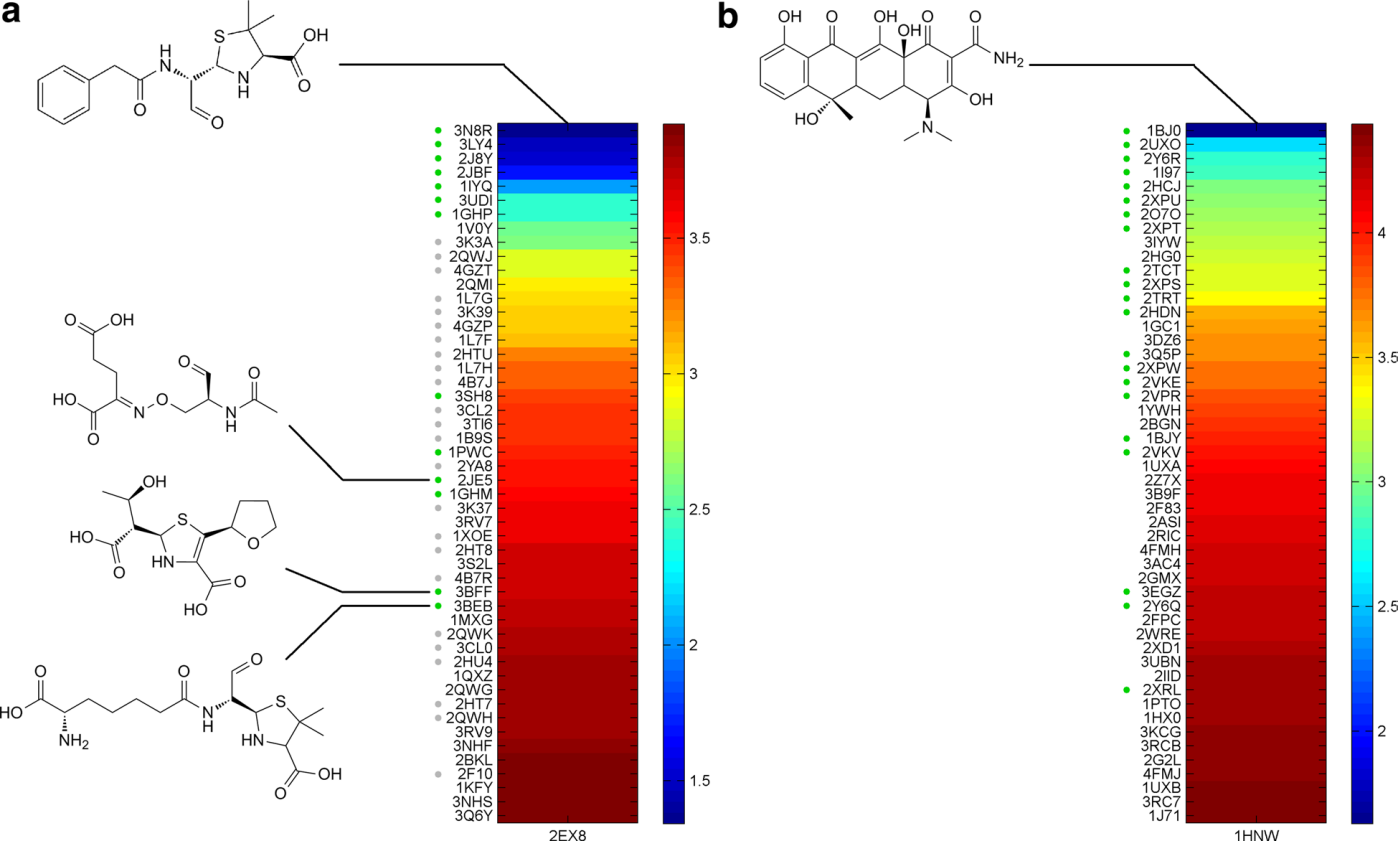
Heat map strips of dissimilarity scores for searching by: **a** open-form penicillin G (*green dots* penicillin binding proteins inhibitors; *gray dots* neuraminidase inhibitors), **b** Tetracycline (*green dots* different tetracycline derivatives bound), corresponding chemical structures are displayed. The *colour legend* represents the dissimilarity score (*d*) scale (see “Methods” section).

**Fig. 3:**
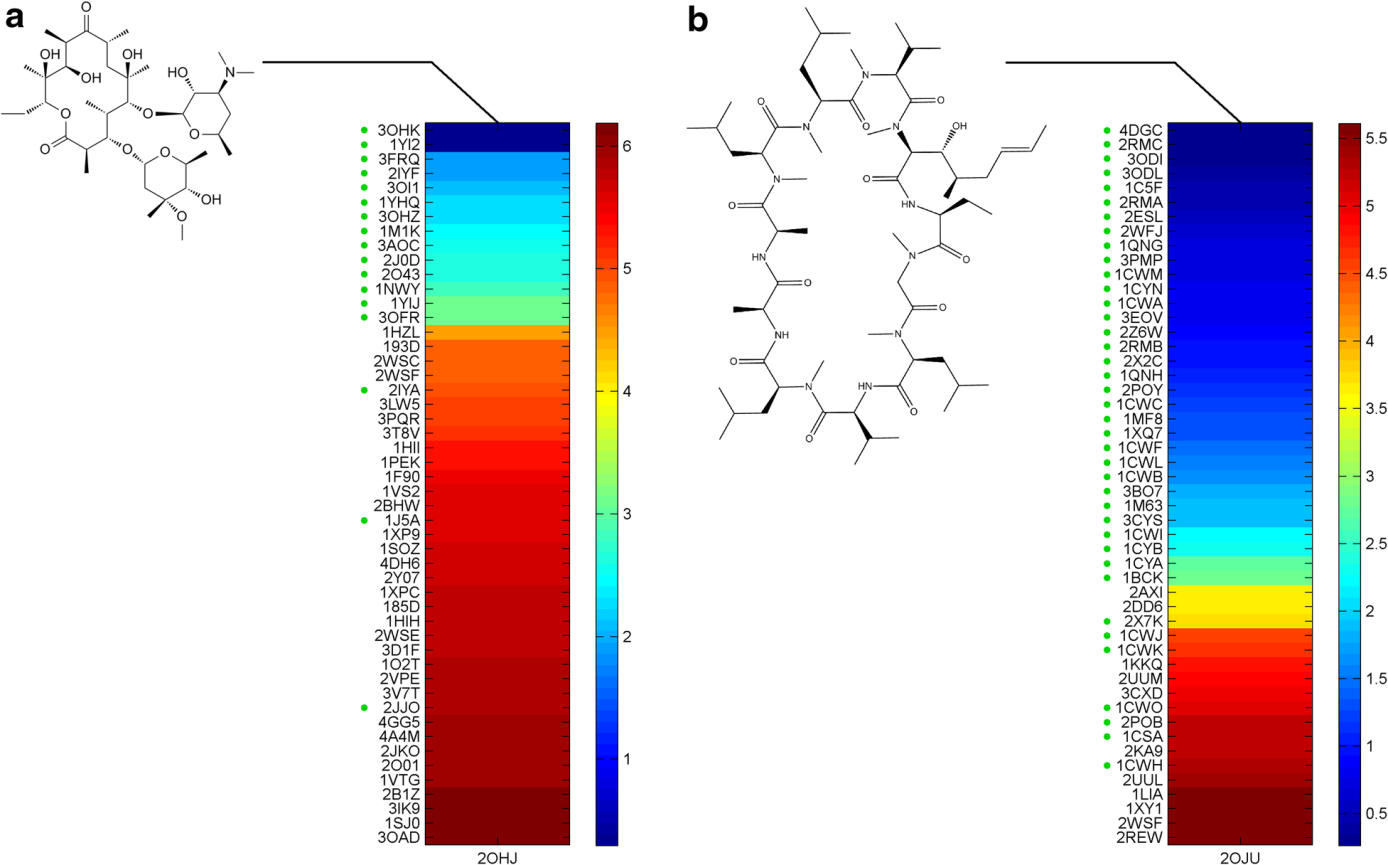
Heat map strips of dissimilarity scores for searching by: **a** erythromycin A (*green dots* different protein synthesis inhibitor macrolides), **b** cyclosporin A (*green dots* different immunosuppressant cyclosporin derivatives). The *colour legend* represents the dissimilarity score (*d*) scale (see “Methods” section).

Then, four more different searches were run using the open-form penicillin G bound to the penicillin binding protein 4 of *E.coli* (PDB: 2EX8) (with and without conformers generation), tetracycline bound to the 30S ribosomal subunit of *T. thermophilus* (PDB: 1HNW), erythromycin A in complex with *T. thermophilus* ribosome (PDB: 3OHJ), cyclosporin A as complexed with human cyclophilin J, and a thiocholine bound to an acetylcholinesterase (AChE) from hydrolysis of butyrylcholine (PDB: 2HA7). The top 50 hits for each query are shown in Figs. 2, 3.

**Fig. 4:**
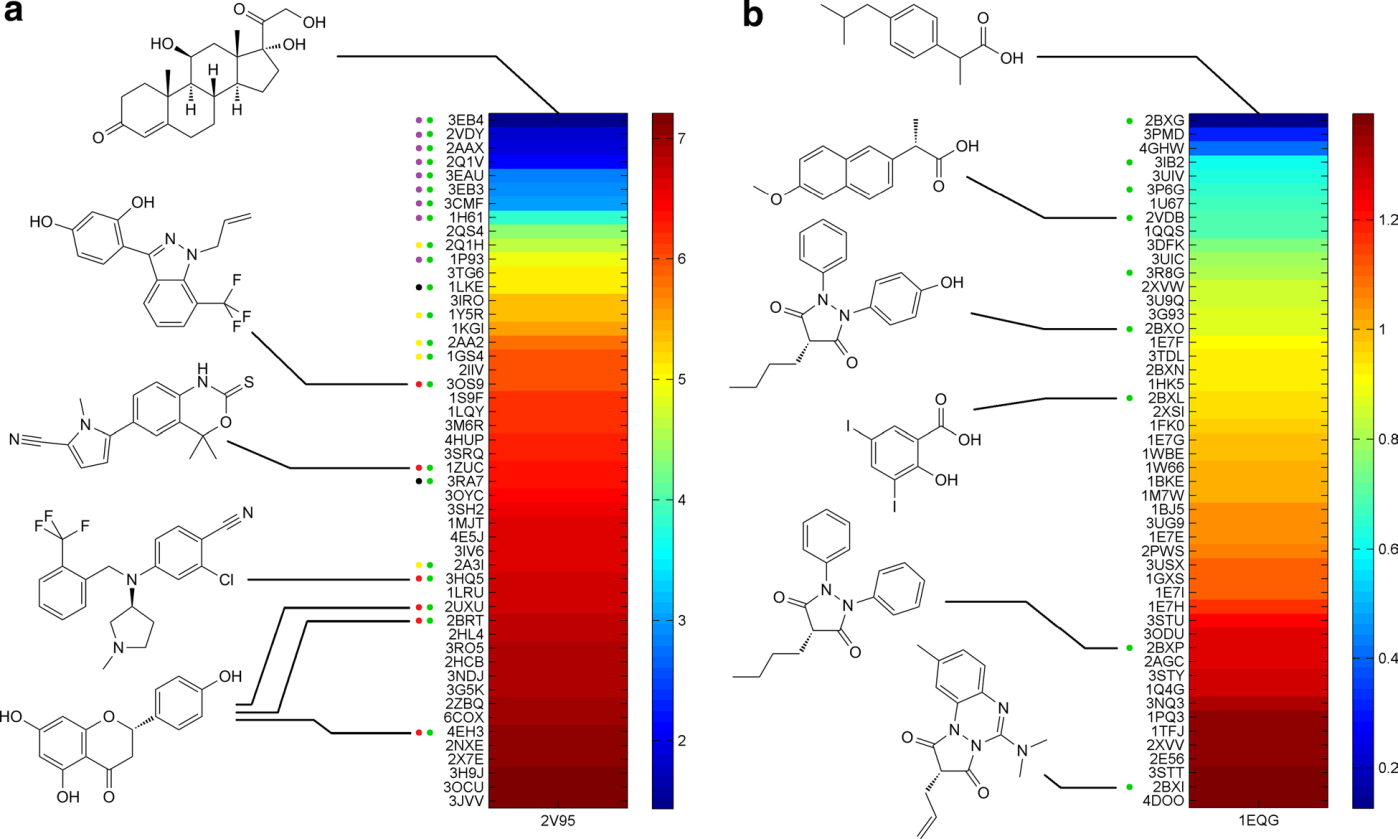
Top search results for searching by: **a** cortisol (*green dots* hits with either steroidal scaffold or steroidal activity; *purple dots* with glucocorticoid activity, *yellow dots* with mineralocorticoid activity, *red* with estrogenic activity, *black* other), **b** ibuprofen (*green dots* NSAID hits). The *colour legend* represents the dissimilarity score (*d*) scale (see “Methods” section).

Finally, and according to the discerned performance variation, two more cases were specially chosen to demonstrate the possible weaknesses of the presented method; namely, cortisol bound to the corticosteroid-binding globulin (PDB: 2V95) and ibuprofen bound to ovine COX-1 (PDB: 1EQG) (Fig. 4).

### Large scale dissimilarity analysis

Although the computation procedurally generates the dissimilarity scores for the full matrix (i.e. 164,939 × 164,939), however, the score arrays were sorted and truncated to the top 100 for each ligand search due to the intractably huge volume of the full-sized matrix, which would imply a volume of 164,939^2^ multiplied by the system’s float variable size. The results were saved as compressed ANSI-encoded files and are provided in the Additional file 1.

### Ligand-based benchmark

As described in the “Methods” section, the enrichment performance of the proposed algorithm was tested against 99 datasets from the DUD-E [10]. The performance was compared to what was reported by Schreyer and Blundell [11] at the 1.0 and 0.25% fractions for USR and USRCAT algorithms. The results showed that the proposed method clearly outperforms both USR and USRCAT at the 0.25% level, and for some targets (marked by asterisks in Additional file 2: Figure S1) all of the retrieved compounds in the top fraction were actives (Additional file 2: Figure S1). Details on datasets sizes, number of conformations sampled, and the individual enrichment factors for each query were reported for each dataset (Additional file 3).

### Binding pocket classification

**Fig. 5:**
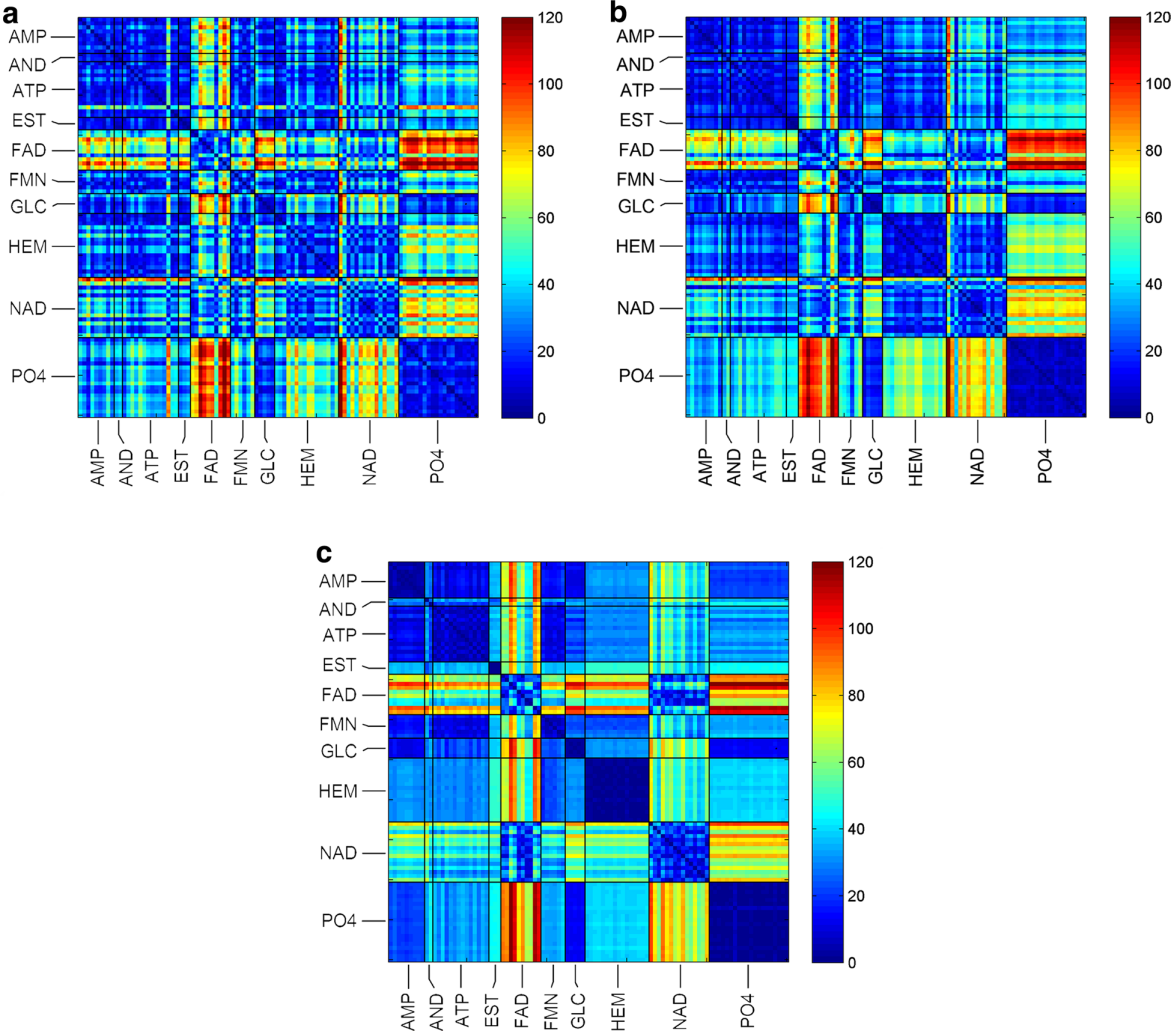
Dissimilarity matrices for the Kahraman benchmark [12]. The *colour code* represented by the legend shows the dissimilarity score value. **a** Pairwise dissimilarity matrix for binding pockets using complete amino acid structures, **b** pairwise dissimilarity matrix for binding pockets using atoms within a 6.5 Å distance from the bound ligand, **c** pairwise dissimilarity matrix for ligand structures.

The Kahraman et al. [12] benchmark is composed of 100 binding pockets for ten ligand groups (originally considered as 9 where the estrogens and androgens are in the same group, but average area under the curve (AUC) values were calculated assuming 9 groups). The cognate binding sites were aggregated from phylogenetically distinct targets (different CATH H-levels). The dissimilarity matrix was generated twice for pocket vs. pocket using a similar but more rigorous threshold, by expanding the binding pocket vicinity atoms to 6.5 Å, which should constitute a more difficult case than selecting a cut-off of 4 or 5 Å. The all-against-all dissimilarity matrices were accordingly generated and the colour legend uniformly levelled at 120 (maxima from the three matrices ranged between 121 and 134) as shown in Fig. 5, while the Additional files section shows the corresponding receiver operating characteristic (ROC) analyses (Additional file 4: Figure S2).

## Discussion

### Ligand definition and charges treatment

Different ligand structure databases adopt different technical criteria for incorporating ligands. For instance, FireDB [13], PDBbind [14], and ProtChemSI [15] exclusively consider organic small molecules, whereas PepX [16] and RsiteDB [17] focus on peptide and RNA ligands, respectively. Binding MOAD [18] and BioLiP [19] offer a wider focus of the ligand chemical nature and molecular weight.

In this study the aim was to achieve maximal comprehensiveness through setting forth very loose criteria for ligand definition (as described in “Methods” section), with an upper bound for the molecular size of 485 atoms. This approximately corresponds to the average molecular size of a triacontameric peptide or an average molecular weight of 3.6 kDa, and is far beyond the generally accepted molecular weight cutoff (500 Da) for oral bioavailability, which in itself is not a hard limit [20]. However, the chemical nature of the analysed structures was not exclusive for peptides, but rather any molecule that fitted the described criteria (and hence a generic charges method was used for partial charges assignment). These criteria resulted in the extraction of 164,939 structures, upon which the analysis was based.

### Molecular topologies

Ligand-based drug design methodologies are based on the assumption that chemical structure similarity is generally linked to biological activity relatedness [21]. This fact formed the basis for development of a wide array of descriptors (chemical, structural, field, pharmacophoric, etc.) which resulted in the proliferation of fast algorithms suitable for virtual high-throughput screening [22]. 2D fingerprints have been so far the most preferable, owing to their computational efficiency [23], with better performance reported for global features fingerprints which better describe the similarity of biological activity profiles, and so capable of scaffold hopping [24].

On this track, the Ultrafast Shape Recognition algorithms (3D structure-based methods) have provided remarkable classification accuracy at comparably low computation costs, the results were similar to the partitioned pharmacophoric shape recognition [11] or charge-inclusive 4-dimensional shape recognition [25]. However, in these methods the distributions construction essentially relies on centroid definitions as discussed in the original publications [25, 26], the mapping of which is highly sensitive to small conformational changes, especially, with large-sized molecules (e.g. side-chain flexibility in a folded peptide). Slight changes in conformations could result in a significantly different centroid mapping, and consequently totally different shape distributions and potential inaccuracy in molecular similarity calculation. Additionally, in the latter method, which treats the atomic partial charge as a fourth dimension in describing the atom position vector, charge scaling must be made, and the way adopted for that was purely a matter of trial-and-error to obtain the best enrichment factors. As a consequence the optimal value of the scaling factor relies on the validation set being used, which might not be an appropriate means if the data set optimal scaling factor happens to be different from the benchmarking set. Lastly, vector normalisation in those methods would contribute to scale invariance as described below, which makes the method unsuitable for handling datasets containing broad size variance.

The proposed algorithm was sought to avoid such drawbacks by detouring centroid definitions and constructing distributions of all possible pairwise interatomic distances, instead of centroid-atom distances. Averting the inclusion of the scaled partial charges as a fourth dimension was done by partitioning atom groups into separate charge-tiers. This approach effectively decreases the computation cost of the distribution sampling from roughly *O*(*n*^2^) to $O\left( {\frac{{n^{2} }}{k}} \right)$, where *n* is the number of atoms, and *k* is the number of charge-tiers.

**Fig. 6:**
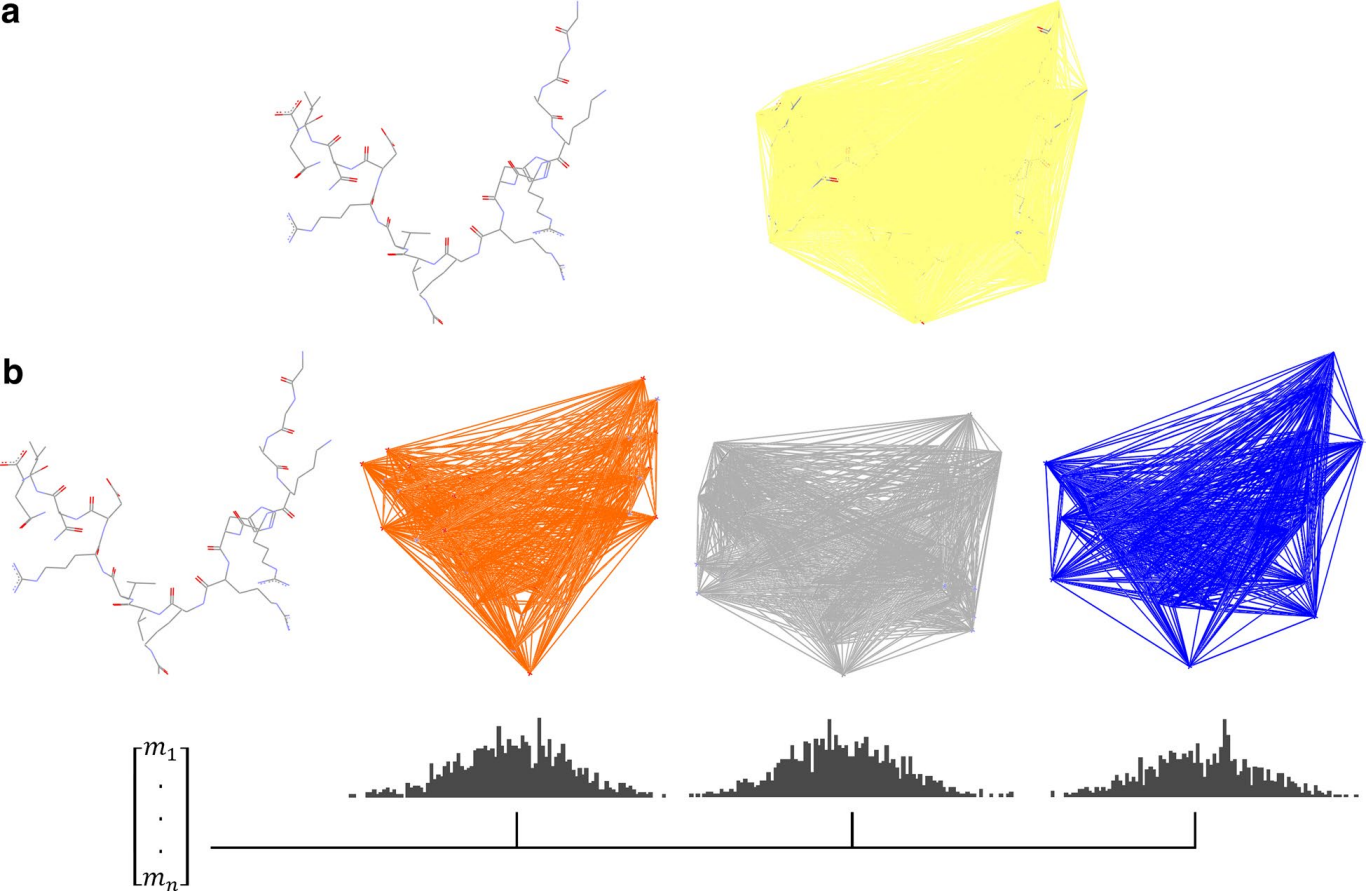
Depiction of descriptor vector construction out of distributions of pairwise distance measurements of a decapentameric peptide (PDB: 2RNY) from all atoms (**a** *yellow* all interatomic distances) versus from three charge-tiers (**b** *red* negative, *grey* non-polar, *blue* positive), hydrogens were removed from display for simplicity.

Since this method is a variant of shape distribution algorithms [27], it has advantages of translational and rotational invariance, and hence very fast topological matching after initial descriptor vectors have been computed. As an illustration, a graphical depiction of the distance measurements was made for a folded conformation of an acetylated truncated (decapentameric) histone peptide (PDB: 2RNY), with global (Fig. 6a) and partitioned (Fig. 6b) distance measurements. As the global distribution sampling would harbour information pertinent only to the overall molecular steric field, it cannot describe any electrostatic details. Therefore, partitioning the atoms into separate charge groups can be described as a form of coarse-grained encoding of the electrostatic field, whereas the charge-tier comprising the non-polar charges interval could implicitly account for the distribution of the hydrophobically interacting molecules, and collectively, they still encode the net steric field.

Another significant advantage is that this approach of partitioning also circumvents the inherent defect of shape distribution methods, which is the propinquity to distribution normality with the increased shape complexity (as is the case with larger molecules), that in turn results in little discriminating power among such shapes. This is because partitioning provides simpler sub-shapes with less dense distance distributions that still span the whole charge-tier spatial coordinates, resulting in more canonical distributions with lesser probability of approaching normality. The higher order charge-tiering schemes (i.e. where *k* > 3) has indeed proven beneficial in discriminating between complex and large structures (e.g. globular protein domains) by resolving the degeneracy through assigning less atoms per charge-tier (data not shown).

This method of describing molecular topologies is sensitive to conformational changes, which was the rationale behind including ligand structural data from the PDB only, as such data would harbour the additional information of the biologically relevant conformer. While profound conformational distortions of the same hashed molecule can result in significant alterations in the overall topology (i.e. significant dissimilarity scores), small conformational changes result in fairly similar topologies (i.e. negligible dissimilarity scores). This is of importance because in a biological reality, a ligand might exhibit similar or different conformational binding modes in different complexes.

As shown in the “Methods” section, the selected distribution descriptors encode distinct features pertaining to the distribution, for *c*_1_ through *c*_5_ would give an account on atomic spatial dispersion; average interatomic distance, statistical dispersion, distribution skewness, and distribution kurtosis, respectively, within each charge-tier. Hence, such elements do implicitly encode a variety of physically translatable features.

### Topology comparison measures

According to the topology encoding method and the use of arrays for canonical molecular indexing, it is noteworthy to emphasise the global descriptive nature of such method. The addition or removal of sufficiently large moieties to the molecular structure would result in a significant change in its descriptors values (and consequently its similarity or dissimilarity scores in relation to reference structures). Subsequently, the method strictly compares global molecular features, rather than local molecular features (substructures), and hence also its conformational sensitivity.

Although using the Manhattan distance as distance measure, the moments composing the vectors were not of the exact value range, with the second and third moments (i.e. *c*_2_ and *c*_3_) being approximately one order of magnitude larger than the other moments. The former indeed physically account for the average distance and spatial dispersion of the corresponding charge-tier atoms, rendering the method more sensitive to the size and extendedness in space, which is a desirable effect, as significant distortions in such parameters (either due to conformational changes or chemical modification) would severely affect the binding capacity of the ligand. Moreover, upon testing the effect of variable and vector normalisation, scale invariance was observed, which was totally undesirable with such a comprehensive definition for ligand inclusion. It might be only tolerated where a narrow range of molecular sizes is allowed, which is the case with handling training sets of actives and decoys, but not with diverse sets.

### Performance analysis

Initial observation of the results shows an excellent enrichment of the topologically relevant hits with obvious scaffold similarity in top ranking portion. The green dots in Fig. 1 (e.g. myricetin, dihydroquercetin, apigenin, kaempferol) could be regarded as the affirmed hits (affirmed true positives owing to the obvious structural similarity). Such subject molecules are complexed with a variety of receptors such as reductase, oxidase, dehydratase, different kinases, and transferases, with a slightly better enrichment by the single conformation of 1H1I as the query structure than by the combined results of the six other conformations. This target diversity is due to the polypharmacological properties of the flavonoids class. However, unobvious topological similarity could be traced in the results. For instance, the result from 1E8W and 1E90 clearly bares a remarkable degree of topological similarity between indirectly relatable chemical classes (flavonoids and nucleotides) [28], which is stressed by the nucleotide-like ligands among the top hits (Fig. 1).

Searching with open-form returned different penicillins and cephalosporins (all in the open-form, owing to the significant topological differences between the open-form and the closed-form). Interestingly, among the top-ranked (topologically similar) hits were molecules of notable structural dissimilarity (Fig. 2a), which were a lactivicin (2JE5), an open-form penem (3BFF), and an incomplete peptidomimetic penicillin structure (3BEB; terminal 2-aminobutanoate coordinates missing from the PDB entry), leading to the assumption that such form of topological similarity is dictated by the compositional conservation of binding cavities among the penicillin binding proteins (which are responsible for conforming the ligands in the corresponding complexes). Another observation was the high propensity of finding neuraminidase inhibitors among the top hits (gray dots in Fig. 2a), which points out a degree of topological similarity to open-form penicillin G. This was consistent with the high rate of penicillins retrieval using oseltamivir analogues as queries (Additional file 1). In order to assess more the effect of conformational distortion on such a highly flexible query molecule, 40 conformations were generated and used as combined queries where the hits were collectively sorted to inspect the dissimilarity minima, which as expected resulted in a much lower hit-rate among the top 50 which is expected due to the 40× expansion of the search results.

A very good enrichment was also observed for tetracycline (Fig. 2b) and the results comprised different tetracycline derivatives bound to different proteins (tetracycline repressors, Tetx monooxygenases, multidrug binding protein, elongation factor Tu, ribosomal subunit and a tetracycline aptamer). Another polyketide, the macrocyclic lactam-based antibiotic, erythromycin A (Fig. 3a) was used as a search query. The latter retrieved different but related macrolides bound to different targets. The retrieved ligands contained erythromythin derivatives as well as other macrolides (clarythromycin, oleandomycin, telithromycin and azithromycin). The hits were bound to different targets including ribosomal subunits, macrolide biosensor proteins, macrolide glycosyltransferases, multidrug exporter protein and cytochrome P450.

In order to demonstrate the effect of the query structure size, a relatively large molecule was used as query. The cyclic non-ribosomal peptide cyclosporin A (a potent immunosuppressant), which is approximately 1.2 kDa, was chosen. The query conformation was of the cyclophillin J bound state. Results (Fig. 3b) show superior enrichment performance compared to other small molecule examples, comprising different modified cyclosporins.

As an additional case where the binding pocket is narrow and the ligand is small, searching with the structure of an AChE-bound thiocholine query structure was performed. The retrieved top hits were enriched with choline derivatives (or mimetics) bound in a very similar (extended) conformation to a variety of specific targets from distant proteins; including acetylcholinesterases, acetylcholine binding proteins, choline transporters, a choline acetyltransferase and a choline kinase (Additional file 5: Figure S3). This may point to the role of specific binding in rigidifying the ligand pose in related binding pockets (in this case, the prevailing feature was the cation-π contact with anchoring hydrogen bond by the hydroxyl/thiol functional group).

As a general observation, score trends appear to be conforming to the enrichment performance, which could be deduced from the number of affirmed hits in the top scoring fractions, as a very high concentration of affirmed hits were found roughly around and below a dissimilarity score value of 4. It was also noticed that sub-structure overlapping hits become more evident with the trendy increase in dissimilarity scores, however, if such sub-structures are of small size proportion, then major topological differences exist with the consequence of an abrupt increase in the dissimilarity score.

In view of the test-cases results, some main factors were noticed to influence the enrichment. Firstly, the molecular size of the query structure, as this would entail more distinct topological features (as long as the distributions are not excessively dense per charge partitioning scheme) which was clearly illustrated by the cyclosporine example. At large molecular sizes the distribution shapes become less sensitive to the relatively small sized chemical modifications, or proportionally subtle conformational changes as compared to major fold alterations. This should be considered inline with the expectation that better enrichment at a particular molecular size is achieved when the query structure atoms are best dispersed between the different charge-tiers; the situation where the intramolecular atomic charge diversity is greatest. Secondly, the relative abundance of the topological classes in the PDB can directly affect the respective number of true positive hits in the top ranking fraction when searching for under-represented topologies. Noteworthy, with the advancement of structural data deposition rates, this should be subdued with time. Thirdly, in case of covalent inhibitors, which do not abide by a well-defined pharmacophore, these are expected to be distantly scored from non-covalently interacting ligands. Finally, in some ligand classes the biological response or target specificity can vary significantly upon very slight chemical modifications, and subsequently, minor topological modifications. Therefore specific analysis methods need to be additionally considered when studying such classes with the described method.

In order to demonstrate this, two special cases are shown. Corticosteroids can provide an example of rigid ligands that can exhibit a very wide range of biological activities upon minor chemical modification of its nucleus. This implies that minor topological changes can significantly alter the receptor specificity. In fact this was evidently observed upon the analysis of the top hits when using cortisol as the query structure (Fig. 4a). The results comprise corticosteroids of diverse biological effects and not just glucocorticoids (i.e. mineralocorticoids, estrogenic compounds, as well as other steroidal topologies), and interestingly, various non-steroidal structures with steroidal activity (modulating different steroidal receptors) were also entrained in the top hits, which further stresses the capacity of the method to identify structurally unrelated topological similarity between different ligands. The other case was ibuprofen, which is a relatively small molecule, and effectively underlines the low accuracy that can originate from sub-structural similarity and the poor level of topological discrimination at such small molecular sizes. This is due to interference from other small molecules with relatively close distribution averages and or their narrow variances. This is evident from the very low dissimilarity scores reported and the relatively poor enrichment performance (Fig. 4b). However, ligands of the same pharmacology with unrelated structures are still among the top hits.

### Ligand-based virtual screening benchmark

To demonstrate the fitness of the proposed method for applications for the purpose of ligand-based virtual screening, the enrichment performance was tested against datasets from the DUD-E [10]. The targets spanned a diverse group of ninety-nine receptors and enzymes of strong pharmacological relevance. The enrichment results were compared to those of the USR [26] and the USRCAT method [11]. Analysing the enrichment performance at a top scoring fraction of 1.0% shows a clearly superior enrichment compared to the USR (higher enrichment factors for 83 out of 99 datasets), which was not the case for USRCAT (higher enrichment factors for 42 out of 99 datasets), albeit that some targets (e.g. *FPPS, SAHH* or *PUR2*) witnessed a great boost in enrichment. More interestingly, was the performance boost for various targets at the 0.25% fraction and the superior enrichment in comparison to both, the USR (higher enrichment factors for 81 out of 99 datasets) and USRCAT (higher enrichment factors for 66 out of 99 datasets). Noteworthy, is the number of cases at the 0.25% fraction where the average enrichment factors were incalculable (using the equation described in the “Methods” section), as some actives as queries retrieved no negative hits in this fraction of top hits (Additional file 2: Figure S1; Additional file 3). This boost in enrichment may be attributed to the aforementioned algorithmic design differences which build on the previous methods weaknesses. This also bares the method’s competence as a practical choice for ligand-based drug design applications, especially, with its demonstrated chemotype-insensitivity, and thus, its capacity for scaffold-hopping design.

### Binding pocket classification benchmark

The demand for protein sequence- and secondary structure-independent methods for mapping binding pockets has risen recently for the purposes of binding site classification, functional relationships predictions, and prediction of pharmacological intersections [29, 30]. To this end, pharmacophore multiplets-based fingerprints have been reported for representing both binding pockets and ligands [31, 32], but which are expected to behave similarly to other pharmacophore-based methods.

It is arguably safe to assume that the binding cleft on a receptor harbours sufficient information to encode the shape complementarity between the ligand and the receptor, and thus, ligand–ligand structural similarity should imply pocket–pocket similarity, and vice versa. However, Kahraman et al. [12] concluded that perfect shape complementarity is not necessarily observed in natural complexes, and that the binding pockets even exhibit, on average, a threefold larger cleft volume than the bound ligands. Therefore, they tried to readjust their spherical harmonic shape signature by normalising their shape coefficients to eliminate size-based interference, and conversely by using zeroth order coefficients to eliminate shape-based interference. These tests were applied to their three models; the Conserved Cleft (fingerprinting restricted to conserved cleft regions shapes), Interact Cleft (fingerprinting restricted to protein atoms interacting with the ligand), and Ligand Cleft (fingerprinting restricted to ligand atoms). Their best cleft vs. cleft performance resulted in AUC values of 0.53 (Conserved Cleft model) and 0.77 (Interact Cleft model). Our models for cleft definition were defined to be more rigorous; in our first model we used all of the cleft residues atoms (including backbone atoms) (Fig. 5a; Additional file 4: Figure S2A), in the second model we used a uniform atom inclusion distance from the ligand of 6.5 Å (which is around 2.6 Å further than HBPLUS interaction distance cut-off) (Fig. 5b; Additional file 4: Figure S2B). Interestingly, our average AUC values are much better, with values of 0.76 and 0.85 for the first and second models, respectively. The results were still better compared to more recent reports of the PSIM method [33] and the Top-3 method [34] which yielded average ROC AUC values of 0.79 and 0.82, respectively.

The superiority of the represented—albeit still a global geometrical fingerprint—to the above discussed spherical harmonic signature could be attributed to the electrostatic (and implicitly the hydrophobic) information encoded in it. Notably, the average AUC values are almost similar between the two methods when comparing ligands (0.94 for the method presented here, and 0.92 for the spherical harmonics fingerprint). This performance similarity for ligands can be attributed to the much simpler topologies in comparison to their binding pockets and the very small size of the benchmarking set, which renders it an easier classification task for a shape-only signature. It can also be observed that in general more rigid molecules tend to be better classified as noted above (Additional file 4: Figure S2C; e.g. FMN vs. FAD, AMP vs. ATP and NAD vs. FAD).

Considering the ligand–ligand similarity as the ground truth (Fig. 5c), we demonstrate the presence of a reminiscent similarity footprint at the pocket–pocket side. The dissimilarity matrices (Fig. 5) bare this proposition and show that the pattern becomes clearer as atoms closer to the interior of the cleft are exclusively fingerprinted (Fig. 5b). This pocket–pocket similarity is that of the ligand’s complementary topology, which is supposed to provide the above-random relationships between internal pocket topologies regardless of their amino acid sequence composition.

## Conclusion

A novel molecular topology comparison method that is based on a combined shape distribution and charge binning scheme was described. The method presented bared the advantages of: generality as to the input chemical structures, capability of handling a wide range of structural sizes, demonstrable utility in scaffold hopping design (which is a merit of ligand-based design), suitability for high-throughput searches (due to the very low CPU-footprint of the calculations involved) and superior performance in mapping of binding cavities.

Although no attempts of tuning the performance of the method for the sake of maximal generality, the descriptor vectors are very easily amenable to machine learning implementation, which is expected to greatly enhance the discrimination power of the method (albeit at the cost of generality and the requirement of a training set). This generality was evidently useful in obviating a clear relation between the pairwise pocket dissimilarity patterns and the corresponding pairwise ligand patterns, which were generated by the exact same implementation. Moreover, the method was able to capture topological relationships between pharmacologically similar but chemically dissimilar ligand classes. These features combined, may allow the use of the described method for a large-scale study of ligand and binding-site promiscuity determinants, and potentially, the prediction of ligand selectivity in prospective scenarios based on topological similarity profiles.

As an outlook from the findings above, we foresee the development of an ultra-fast implementation of a 3D local topological mapping (as opposed to the global mapping described) and surface-restricted sampling, for the purpose of capturing sub-structural relations and acquiring only the surface-laden information (which is the most pertinent to the molecular recognition events), respectively.

## Methods

### PDB data treatment and structure preparation

A snapshot of all of the Protein Data Bank entries (the 1st of January-2013 release) was obtained from the relevant ftp server (ftp://snapshots.wwpdb.org/) which amounted to 87,090 PDB coordinates files.

The coordinates were then processed by removing all records pertaining to molecules comprised of less than 10 atoms (including solvent atoms and monoatomic ions), and for the sake of charge treatment generality, organometallic complexes were stripped of their metallic cores. For multi-model PDB files, the first model was extracted as the representative of the corresponding entry coordinates. The upper cut-off for the ligand number of atoms was defined as 485 atoms regardless of its chemical nature.

Because of the heterogeneous chemical nature of the processed molecules, the atomic partial charges were computed using a general charges method; hydrogens were added to the structures at a pH of 7.4 and Gasteiger charges were assigned using Open Babel [35]. The generation of conformations in some test-cases was carried out using Confab [36]. The conformation sampling procedure used an RMSD cutoff of 0.65 Å, energy cutoff of 35 kcal/mol, and a maximum of of 5,000 sampled conformers while keeping the input conformer.

### Topological computations and fingerprinting

The Python programming language [37], and the NumPy and SciPy packages [38] were used to implement the algorithm of molecular topology fingerprinting, which consisted of the following main steps: (1) partitioning the molecular coordinates into different charge-tiers, i.e. each of the latter contains a substructure of atoms belonging to its partial charge interval; (2) pairwise shape distribution matrices are computed within each charge-tier; (3) a vector was constructed for each molecule, the components of which are descriptors of the respective shape distributions.

In the first step, after inspecting the full propensity distribution of atomic charges of all the ligand atoms, tripartite partitioning was performed among three charge-tiers. The intervals net charge bounds were selected as: greater than +0.1, smaller than or equal +0.1 and greater than or equal −0.1, and smaller than −0.1. These intervals lead to a reasonably symmetrical population of the charge-tiers (to avoid charge bias).

The fifteen elements composing a descriptor vector; five distribution descriptors of each of the three distributions (of each charge-tier), were calculated as follows:

$$c_{1} = \frac{{\mathop \sum \nolimits_{i = 1}^{n} \mathop \sum \nolimits_{j = 1}^{n} x_{ij} }}{{n^{3} }}$$

$$c_{2} = \overline{x}_{ij}$$

$$c_{3} = \frac{1}{{n^{2} }}\mathop \sum \limits_{i = 1}^{n} \mathop \sum \limits_{j = 1}^{n} (x_{ij} - \overline{x}_{ij} )^{2}$$

$$c_{4} = \frac{{\frac{1}{{n^{2} }}\mathop \sum \nolimits_{i = 1}^{n} \mathop \sum \nolimits_{j = 1}^{n} (x_{ij} - \overline{x}_{ij} )^{3} }}{{(m_{3} )^{3/2} }}$$

$$c_{5} = \frac{{\frac{1}{{n^{2} }}\mathop \sum \nolimits_{i = 1}^{n} \mathop \sum \nolimits_{j = 1}^{n} (x_{ij} - \overline{x}_{ij} )^{4} }}{{(m_{3} )^{2} }} - 3$$

where *x*_*ij*_ is the atomic pairwise distance between the *i*th and the *j*th atoms in the distances matrix (skipping the diagonal points; where $i = j$), *c*_2_ is the sample mean, *c*_3_ is the sample variance, *c*_4_ is the distribution skewness, while *c*_5_ is the distribution Fisher kurtosis. Zero- or one-atom-tiers had their corresponding moments elements valued to zero. Although the contribution of some elements to the inter-vector distances is much greater than others (which is also influenced by general structural features of the dataset), all the described elements were kept for their potential utility in combination with weighting factors (e.g. in a machine learning context).

### Dissimilarity quantification

Since the distribution descriptors information was loaded into a vector form, various vector distance measures could provide a quantitative estimate. Although the moments’ values were not of the same unit, neither vector nor variable normalisation was adopted (due to reasons in the discussion section), and for comparing descriptor vectors the Manhattan distance was used as follows:

$$d\left( {\varvec{v}_{1} , \varvec{v}_{2} } \right) = \mathop \sum \limits_{i = 1}^{n} |v_{1i} - v_{2i} |$$

where *d* is the dissimilarity score between the vectors $\varvec{v}_{1}$ and $\varvec{v}_{2}$, which was intended in order to give equal weights to all of the moments composing a vector. This gave better performance than the Euclidian distance, but also provides a linear response upon vector element reweighting (e.g. in machine learning applications). Alternatively, clustering approaches could be used, which can be optimised to give better results, albeit on the expense of the computation cost.

A testing implementation of the described method was made available (Additional file 6).

### Test cases

Seven different examples were selected for assessing the capacity of the method of detecting ligand-based relationships and to demonstrate optimal search influencing factors. In order to explore diverse chemical examples of high pharmacological importance, three of which were small molecules, two were chosen to be of peptide nature, while the remaining two were particularly chosen to demonstrate the conditional inferential weaknesses of the method. The examples were: quercetin, open-form penicillin G, tetracycline, erythromycin A, cyclosporine A, cortisol, and ibuprofen.

### Ligand-based virtual screening benchmark

The directory of useful decoys-enhanced (DUD-E) [10] was used for benchmarking sets. The dataset was comprised of ninety-nine targets of different natures, for which enrichment results were already reported for the USR and USRCAT algorithms [11].

A more rigorous conformational sampling scheme was used for both actives and decoys, with an RMSD cutoff of 1.1 Å, an energy cutoff of 50 kcal/mol, and a maximum of 10,000 sampled conformers while keeping the input conformer. The lowest energy conformer of each active was identified through the obenergy application from the OpenBabel suite [35]. The search conditions enrichment factor calculation procedure were chosen to reproduce those described for the previous benchmarking [11]; through conducting the searches using the lowest energy conformer of each active, while excluding the query active from the results, followed by the calculation of the averaged enrichment factor for all actives. No charge assignment was performed since charges were already reassigned by the Confab software. The individual enrichment factor was calculated using the previously described equation:

$$EF_{i,x\% } = \frac{{a_{i,x\%}}/{d_{i,x\%}}}{A/D}$$

where *a*_*i*,*x*%_ is the number of actives retrieved in the top *x*% fraction of the search results using the *i*th active as query, *d*_*i*,*x*%_ is the number of decoys retrieved in the same fraction, while *A* and *D* are, respectively, the total numbers of unique actives and decoys in the dataset of a single target. The averaged enrichment factors for each target were calculated at *x*% fractions of 1.0 and 0.25%.

### Binding pocket classification benchmark

The exact same procedure for data pre-processing, hashing and dissimilarity measurement was followed. The structures were handled using the same partial charges treatment and the same charge-tier scheme for the case-testing above. The ROC analyses were conducted as described in the reference study [12]. Three dissimilarity matrices were generated, pocket vs. pocket using all binding site atoms (of every amino acid within a 5 Å distance from the ligand), pocket vs. pocket using binding site atoms only within 6.5 Å of the ligand, and inversely, ligand vs. ligand using the ligand atoms only.

### Construction of a reduced dissimilarity matrix

Python scripts were written for preparing and staging a batch job on the Hydra supercomputing facility with the aim of generating a reduced pairwise dissimilarity matrix (for all of the ligands included in the analysis) with the dimensions of 100 × 164,939, i.e. computing the top 100 hits resulting from a PDB-wide search using each ligand as query.

## Additional files

Additional file 1: Results large scale dissimilarity analysis. Top 100 dissimilarity scores for each ligand search saved as split-compressed ANSI-encoded file. File available at https://github.com/ElGamacy/PDB-ligands. Additional file 2:
**Figure S1.** Enrichment factors for 99 DUD-E datasets as resulted from the presented method (TopMap) and reported from the USR and USRCAT methods [11]. The left and right panes show the average enrichment factors for each target at top scoring fractions of 1.0% and 0.25%, respectively. Asterisks indicate that one or more actives retrieved no decoys in the top scoring fraction by TopMap (rendering the described enrichment factor calculation inapplicable). Additional file 3: A tar archive containing the details of the enrichment results, including the individual query (uniquely retrieved actives and decoys at the 1% and 0.25% top fractions), averaged enrichment factors for all of the sets, number of unique ligands and decoys and the number of generated conformers. Additional file 4:
**Figure S2.** Receiver operating characteristic (ROC) plots showing classification true positive rates *vs.* false positive rates and the corresponding area under the curve (AUC) values for Fingerprinting: **A.** using all binding pocket residues atoms, **B.** using binding pocket residues atoms within 6.5 Å from the ligand, and **C.** using ligand structures. Additional file 5:
**Figure S3.** Top search results for searching by: Thiocholine bound to AChE (green dots: hits with choline or choline-mimicking scaffold; blue dots: bound to AChE proteins, red dots: bound to acetylcholine-binding proteins, yellow dots: bound to acetylcholine transporters, black: other). The colour legend represents the dissimilarity score scale. Additional file 6: Linux and Windows builds of a testing implementation are provided along with walk-through test-usage instructions on an exemplary fingerprinting and dissimilarity calculation. The builds and instructions are available on https://github.com/ElGamacy/TopMap-SD.
