# Supplementary figures and images for: A fast topological analysis algorithm for large-scale similarity evaluations of ligands and binding pockets
Source: J Cheminform. 2015 Aug 20;7:42. doi: 10.1186/s13321-015-0091-5 (PMC4631714; doi:10.1186/s13321-015-0091-5)

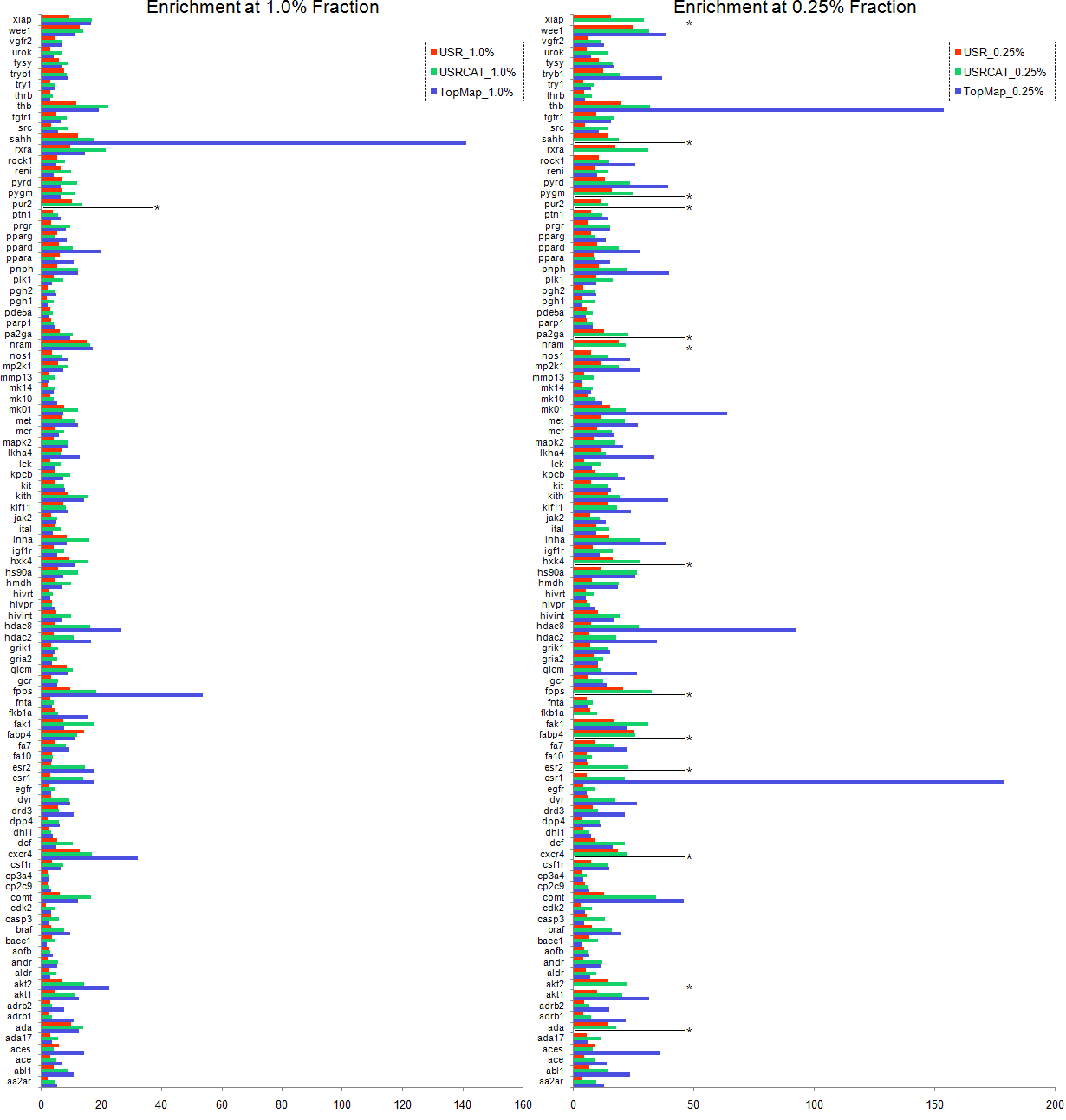

Supplement: Additional file 2: — Figure S1. Enrichment factors for 99 DUD-E datasets as resulted from the presented method (TopMap) and reported from the USR and USRCAT methods [11]. The left and right panes show the average enrichment factors for each target at top scoring fractions of 1.0% and 0.25%, respectively. Asterisks indicate that one or more actives retrieved no decoys in the top scoring fraction by TopMap (rendering the described enrichment factor calculation inapplicable). [file 13321_2015_91_MOESM2_ESM.png]

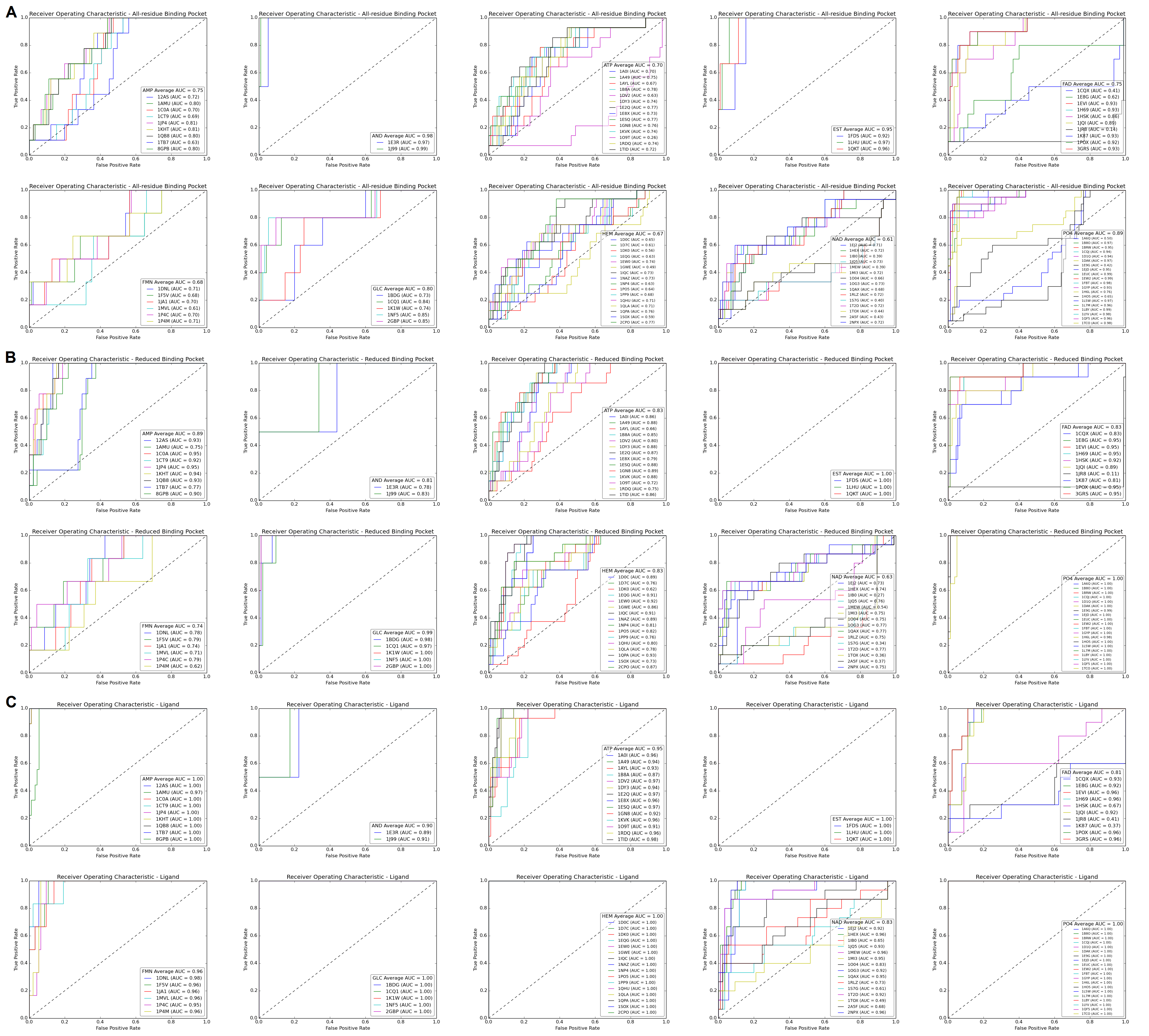

Supplement: Additional file 4: — Figure S2. Receiver operating characteristic (ROC) plots showing classification true positive rates vs. false positive rates and the corresponding area under the curve (AUC) values for Fingerprinting: A. using all binding pocket residues atoms, B. using binding pocket residues atoms within 6.5 Å from the ligand, and C. using ligand structures. [file 13321_2015_91_MOESM4_ESM.png]

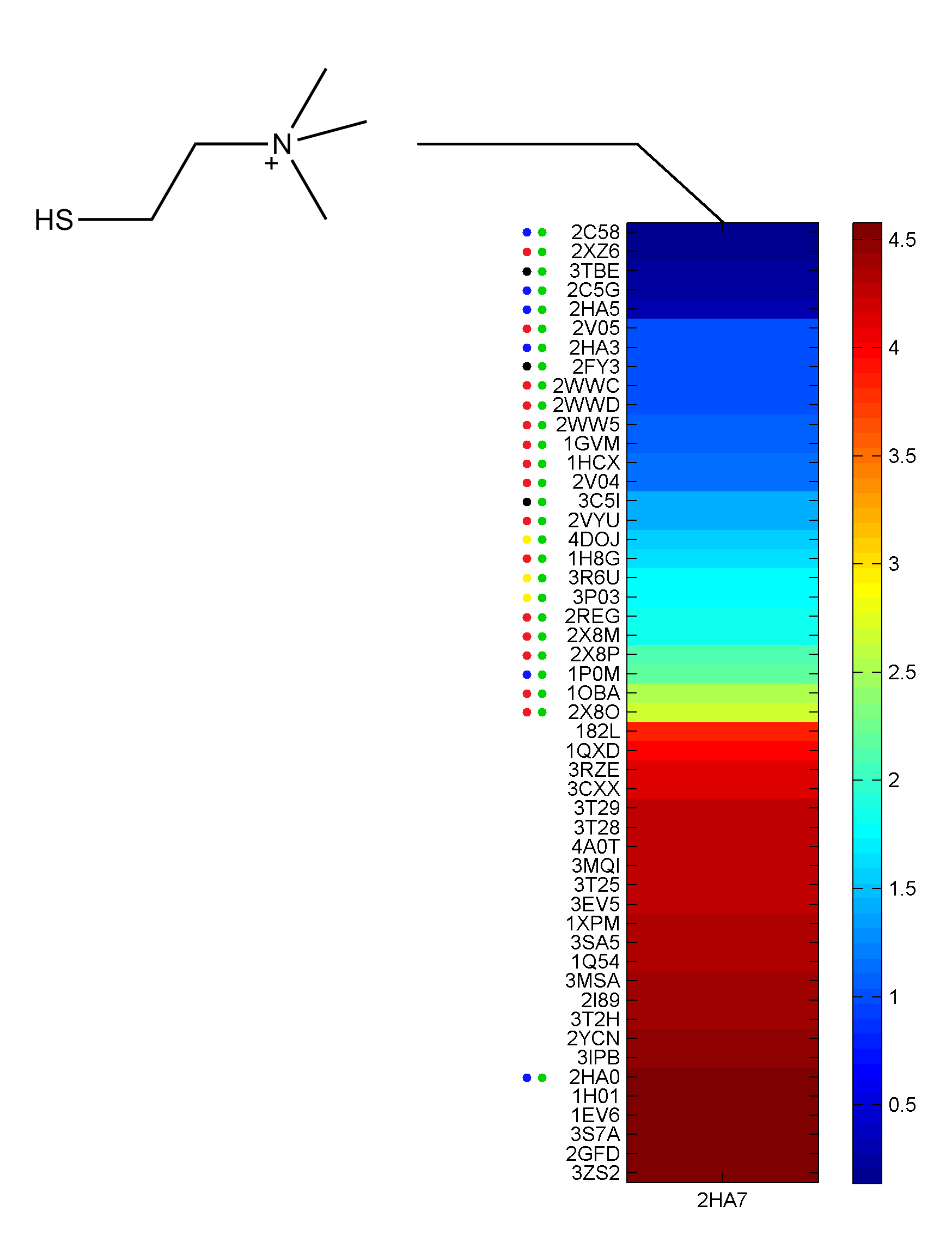

Supplement: Additional file 5: — Figure S3. Top search results for searching by: Thiocholine bound to AChE (green dots: hits with choline or choline-mimicking scaffold; blue dots: bound to AChE proteins, red dots: bound to acetylcholine-binding proteins, yellow dots: bound to acetylcholine transporters, black: other). The colour legend represents the dissimilarity score scale. [file 13321_2015_91_MOESM5_ESM.png]
